# Fighting Tuberculosis: In Search of a BCG Replacement

**DOI:** 10.3390/microorganisms11010051

**Published:** 2022-12-23

**Authors:** Nonna I. Nadolinskaia, Maria S. Kotliarova, Anna V. Goncharenko

**Affiliations:** Bach Institute of Biochemistry, Fundamentals of Biotechnology Federal Research Center, Russian Academy of Sciences, 119071 Moscow, Russia

**Keywords:** BCG, tuberculosis, vaccine

## Abstract

Tuberculosis is one of the most threatening infectious diseases and represents an important and significant reason for mortality in high-burden regions. The only licensed vaccine, BCG, is hardly capable of establishing long-term tuberculosis protection and is highly variable in its effectiveness. Even after 100 years of BCG use and research, we still cannot unequivocally answer the question of which immune correlates of protection are crucial to prevent *Mycobacterium tuberculosis* (Mtb) infection or the progression of the disease. The development of a new vaccine against tuberculosis arises a nontrivial scientific challenge caused by several specific features of the intracellular lifestyle of Mtb and the ability of the pathogen to manipulate host immunity. The purpose of this review is to discuss promising strategies and the possibilities of creating a new vaccine that could replace BCG and provide greater protection. The considered approaches include supplementing mycobacterial strains with immunodominant antigens and genetic engineering aimed at altering the interaction between the bacterium and the host cell, such as the exit from the phagosome. Improved new vaccine strains based on BCG and Mtb undergoing clinical evaluation are also overviewed.

## 1. Introduction

Tuberculosis disease (TB), which is caused by *Mycobacterium tuberculosis* (Mtb), is one of the major reasons of death around the world. A total of 1.6 million people died from TB in 2021 (WHO). This disease is characterized by slow progression; after pathogen penetration and primary infection, latent TB usually develops. In this case, the bacteria are confined in granulomas under immune control to restrict bacterial spreading [1]. When the pressure of the immune system wanes, the disease can develop. About ¼ of the population are carriers of Mtb. In about 10% of cases, TB goes from a latent to an active form when the trabeculae can mature and rupture, releasing free Mtb into the airways for its aerosol transmission. 

BCG (Bacillus Calmette–Guérin), the only TB vaccine available today, provides a certain level of defense, especially in children, but it has some drawbacks as well. Advanced variants of the vaccines offering increased immunogenicity and alternative vaccines based on other species of mycobacteria have long been under development all over the world.

Whole-cell vaccines, especially live ones, appear to be preferred to subunit vaccines [2]. Their advantages include, firstly, their broad antigenic composition, similar to that of the pathogen, which cannot but increase protection. Secondly, BCG and resembling vaccines have also been shown to cause a heterologous immune response called “trained immunity”, which is more difficult for subunit vaccines to promote. Further, the overall positive effect of BCG on immunity in children, which reduces mortality, must be taken into account. Finally, this vaccine is used in the treatment of non-TB diseases, such as bladder cancer, and some evidence also points to the positive contribution of BCG persistence in the organism to the relief of COVID-19.

Due to the emergence of new methods of genetic engineering, the improvement of BCG has received a second wind. Numerous variants of new recombinant vaccine strains and subunit vaccines are being actively developed. 

This review first covers some aspects of the interaction of mycobacteria with the host cells. A discussion of some popular strategies for modifying BCG to enhance its vaccine properties follows, and attention is given to the most successful vaccine candidates that are currently in clinical trials.

## 2. Immune Response to Mycobacterium Tuberculosis

Immune response to Mtb comprises different mechanisms of the innate and adaptive immune response. A characteristic feature of Mtb is its ability to live inside professional phagocytes, which normally eliminates the captured bacteria and activates cells of adaptive immunity. In this connection, the Mtb–host cell interaction greatly affects both the disease progression and immune response. These points are important for understanding the action of vaccines, especially live vaccines as BCG. 

### 2.1. Interaction of Mycobacterium Tuberculosis with A Host Cell

**Figure 1:**
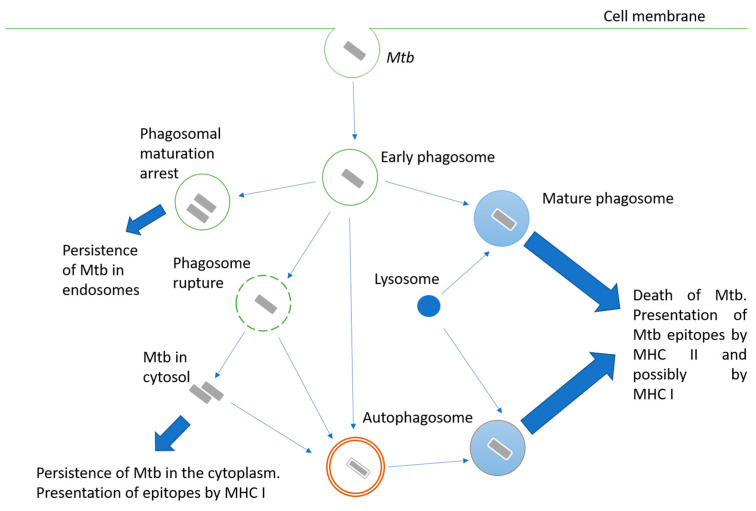
Intracellular fate of a bacterium engulfed by a phagocyte. Description is in the text.

Mycobacteria are intracellular pathogens; they invade alveolar epithelial cells, macrophages, and other phagocytes. When phagocytes engulf Mtb, the mechanisms aimed at destroying the pathogen are triggered, but Mtb may interfere with them and use the engulfing cell as a habitat. Possible options for the fate of the engulfed mycobacterium are shown in Figure 1. The successful maturation of the phagosome usually results in the death of the bacteria. The main antimicrobial mechanisms of the macrophage mature phagosome include luminal acidification, the production of antimicrobial peptides, the production of reactive oxygen and nitrogen species, and, finally, the degradation of enzymes such as cathepsins [3]. Peptides are processed in phagosomes for their presentation in complex with MHC II [4]. Additionally, cross-presentation by MHC I is possible [5].

Inhibiting phagosome maturation in macrophages ensures the efficient intracellular multiplication of mycobacteria, especially at the beginning of infection. Mtb products involved in phagosome maturation arrest include cell wall components such as lipoarabinomannan (LAM) [6], glycolipid trehalose-6,6’-dimycolate (TDM; cord factor) [7], and proteins such as protein kinase PknG [8], phosphatase SapM [9], zinc metalloprotease Zmp1 [10], and urease UreC [11].

Another pathway for Mtb survival in the phagocyte cell is the destruction of the phagosome membrane and its exit into the cytoplasm [12]. The main effector of this process is the ESAT-6 protein, a pore-forming protein secreted via the ESAT-6 secretion system (ESX-1). Therefore, BCG and other mycobacteria, carrying attenuating mutations at the ESX-1 locus, are unable to penetrate the cytoplasm. The penetration of bacterial proteins into the cytoplasm activates their processing for presentation by MHC class I [13].

Since Mtb is able to avoid death in the phagolysosome, autophagy plays an important role in bacterial elimination. Autophagosomes are two-membrane structures surrounding cell components or intracellular pathogens [14]. The fusion of lysosomes with autophagosomes provides an acidic environment for hydrolases, including cathepsin D, oxidases, inducible NO synthase, and antibacterial peptides to kill Mtb and degrade it. Thus, autophagy functions as a second line of defense against pathogens that overcome phagosome-mediated destruction in phagocytes. However, Mtb has developed defense mechanisms against it as well. For instance, ESAT-6 and secreted acid phosphatase SapM have been shown to block autophagosome and lysosome fusion in macrophage-like Raw cells 264.7 [15,16].

An important consequence of mycobacteria entering the cytosol of the host cell is their interaction with cytosolic innate immunity receptors, leading to the activation of the inflammasome [17]. Inflammasomes are complex intracellular structures composed of several proteins that include cytosolic receptors such as AIM2-like receptors (ALRs) or NOD-like receptors (NLRs), adaptor proteins, and effector proteins. Classically activated inflammasomes cleave procaspase 1, converting it to active caspase 1. Caspase 1 carries out the processing and maturation of the important proinflammatory cytokines IL-1β and IL-18 [18].

Cytokines of the IL-1 family play a critical role in the protection against TB. IL-1R knockout in mice led to early lethality after aerosol low-dose infection with Mtb [19]. At the same time, inflammasome dysfunction was found to have less of an impact, which indicates that caspase 1-independent pathways for IL-1β activation can be implemented in TB [20]. 

The mechanisms of cell death following Mtb infection have different consequences for the host. In classical necrosis, the affected cell swells and bursts, causing acute inflammation. The leakage of cell content during necrosis promotes the spread of bacteria [21]. Apoptosis is a generally caspase-mediated cascade of reactions that result in cytoskeleton collapse, DNA fragmentation, the disassembly of cellular components, and packaged cell content into membrane-surrounded apoptotic bodies. There are at least three ways to trigger apoptosis: the interaction of some members of the TNF family with their receptors, cellular stress leading to the disruption of the outer mitochondrial membrane, and the action of granzyme B released by cytotoxic cells. It is generally accepted that apoptosis is an effective defense mechanism against Mtb [22]. Cell apoptosis promotes bacterial death, and apoptotic bodies containing bacteria can be taken up by other antigen-presenting cells and are presented in MHC I by a cross-presentation mechanism [21].

### 2.2. Adaptive Immunity to Mtb

Adaptive immunity is important for the containment of the infection. It is mainly mediated by T-cells. The activation of naive T-lymphocytes occurs in lung-draining lymph nodes where dendritic cells and, to a lesser extent, macrophages locate and present antigens as processed epitopes in complex with MHC I or MHC II [23]. The main effector cells of adaptive immunity against TB are CD4 T-lymphocytes. Mice deficient in these cells are significantly more susceptible to infection than wild-type or CD8 T-cell-deprived mice [24]. When CD4 T-cell deficiency develops due to HIV, the possibility of TB activation dramatically increases as well as the severity of the disease [25]. CD8-lymphocytes are also involved in the immune response to Mtb [26]. Their functions include IFN-γ secretion and cytotoxic action on the infected cells.

Specific CD4 T-cells can form different populations. Th1 lymphocytes are critical as major producers of IFN-γ, TNF-α, and IL-2. The genetic dysfunction of IFN-γ leads to the high sensitivity of mycobacteria [27]. The cytokines IFN-γ and TNF-α promote the activation of infected phagocytes and intracellular elimination of bacteria; they induce autophagy, whereas Th2 cytokines such as IL-4 and IL-13 inhibit it, thereby interfering with the autophagic elimination of the intracellular pathogen [14]. Th17 cells and their main effector, cytokine IL-17, are also important in immune response and may ensure IFN-γ-independent protection against Mtb [28]. Th17 cells provide protection against Mtb infection in adoptive transfer models in IFN-γ-deficient mice, which was reflected by a greater survival and lower bacterial burden. Finally, regulatory T-cells (T reg) secrete IL-10 and TGF-β, which suppress immune response activity and thus can protect tissues from excessive inflammation. During the initial T-cell response to Mtb infection, Mtb induces T reg cell expansion, which delays the onset of adaptive immunity, allowing Mtb to replicate in the lungs until T cells finally arrive [29].

There is no unequivocal opinion on the role of humoral immunity in the protection against the development of TB. It is known that patients with a defective antibody production mechanism and/or patients deficient in B-lymphocytes are not particularly at risk of TB infection, and passive immunization provides no protection. However, several groups have found that monoclonal antibodies against certain cell wall components can modify various aspects of mycobacterial infections [30]. Thus, the immune response to Mtb involves a complex interaction of innate and adaptive immunity mechanisms.

### 2.3. Immune Response and Protection Induced by BCG Vaccination

The only vaccine currently used in clinical practice is BCG. It was obtained through the long-term cultivation of pathogenic *Mycobacterium bovis* in poor media, leading to the loss of several gene loci. The latter are known as regions of difference (RD) and include some sequences that are important for virulence (primarily RD1). A number of other gene rearrangements occurred during subsequent cultivation, resulting in the appearance of different sub-strains, which have some differences in the level of protectiveness and residual virulence [31].

The BCG vaccination is administered in infancy in high-burden countries. BCG protection from disseminated TB and tuberculous meningitis, the two most severe forms of TB in children, is reliably proven [32]. In addition, the effect of BCG on nonspecific immunity and resistance to other infections has attracted a lot of interest lately [33]. For instance, BCG vaccination significantly reduces overall infant mortality [34]. A possible reason for this is the stimulation of nonspecific immunity, resulting in enhanced resistance to respiratory diseases [35]. Other nonspecific effects of BCG include an increase in antiviral immunity through the intensified production of IL-1B [36] and IFN-γ [37]. The immunostimulatory properties of BCG have also found use in the therapy of nonmuscle-invasive bladder cancer with a high risk of recurrence or progression [38]. BCG reveals the anti-tumor activity and is considered the gold standard of treatment for this type of tumor [39]. Its action is based on the recruitment of CD4+ T-cells, neutrophils, and lymphocytes and the activation of immune cells to eliminate cancer [40]. An important mechanism of the nonspecific effect of BCG in the immune response to this one and several other diseases is heterologous immune memory, which is mediated by epigenetic reprogramming and is stimulated by BCG. This phenomenon is called “trained immunity” [41].

An analysis of current epidemiologic data on COVID-19 infection revealed a correlation between BCG vaccination and decreased morbidity and mortality from COVID-19 worldwide [42]. It was found that countries not practicing BCG vaccination, such as Italy, the Netherlands, and the USA, suffered more from the pandemic than countries with common BCG vaccination policies. Countries with late BCG vaccination put into clinical practice, such as Iran (1984), suffered from a higher mortality rate. This supports the hypothesis that BCG is effective in protecting the elderly population. BCG vaccination has been shown to provide broad protection against viral infections and sepsis [33], so it is likely that the protective effect of BCG may not be directly related to protection against COVID-19. Nevertheless, due to the correlation between BCG vaccination and a mortality decrease in reported COVID-19 cases, there is reason to believe that BCG may provide some protection directly against COVID-19. 

The intradermal BCG vaccination of infants has been found to produce functions specific to mycobacteria CD4 and, to a lesser extent, CD8 cells [43]. BCG protectivity decreases with age despite the fact that healthy, BCG-vaccinated children of different ages produce effector CD4+ T-cell responses. This may be due to a weakening of BCG-specific proliferation and the expansion of effector CD4 T-cells with age, which is associated with an increase in regulatory T-cells. [44]. In addition, normal effector T-cell responses, as determined by IFN-γ production, do not necessarily correlate with protective immunity [45].

BCG vaccination can protect against infection with Mtb. The meta-analysis of six studies (n = 1745) showed the effectiveness of BCG-induced protection against infection was 27% compared with 71% against active TB. Among those infected, protection against disease progression was 58%. The effectiveness of the protection against infection among vaccinated children based on 14 studies was 19% compared to unvaccinated children [46].

A key problem with BCG is its varying efficacy. Additionally, BCG has little or no effect on the spread of pulmonary TB in adults. Geographic location, prior exposure to nontuberculous mycobacteria, and the rate of attenuation of BCG are considered factors that influence the effectiveness of BCG [47]. Thereby, scientists all around the world have been developing new vaccines that could provide more effective protection. An increased understanding of the mechanisms of immune defense against TB and the development of genetic engineering methods have allowed the creation of a large number of candidate vaccines that are considered a potential replacement for BCG.

## 3. Strategies to Advance BCG

One promising strategy for improving BCG appears to be the introduction of TB antigens whose expression will enhance the immune response. To date, quite a few antigens have been described that can be used for this purpose.

### 3.1. ESX-1 Secretion System

The attenuation of BCG is caused by the loss of several regions of difference in its genome. The most important of them is RD1, which contains genes encoding ESX-1 components. The major proteins of this system, ESAT-6, and CFP-10, are important Mtb virulence factors that are secreted into the cytosol [48,49]. A study [50] determined which RD1 gene expression products played a role in antigen secretion and, thus, mycobacterial virulence. The most important proteins for the release of ESAT-6 and CFP-10 factors into the cytosol are encoded by the *pe35* (*Rv3872*), *esxB* (*Rv3874*), which encodes CFP-10, and *esxA* (*Rv3875*), which encodes ESAT-6. The other significant genes are *Rv3868, Rv3869, Rv3870, Rv3871*, and *Rv3877*, which encode proteins critical for CFP-10 and ESAT-6 secretion, including chaperone-like proteins that contain an ATP-binding site and probable membrane proteins. They appear to be involved in the formation of the transmembrane complex that enables ESAT-6 and CFP-10 translocation. Extended RD1 also contains several genes, such as *Rv3865* and *Rv3866*, the inactivation of which has not resulted in the damaged expression or impaired secretion of ESAT-6 and CFP-10. Their exact role remains unknown; perhaps they contribute to Mtb virulence independent of the ESAT-CFP secretion system.

These important antigens can be used to create new recombinant vaccine strains (rBCGs). For example, the immune response to the rBCG strain expressing the ESAT-6 secretory antigen, both humoral and cellular, was higher than that of BCG in a mouse model [51]. Many prototype subunit vaccines are also based on these antigens [52,53].

ESAT-6 is often used in vaccines in combination with other antigens, such as Ag85B. For instance, rBCG-AEI expresses a chimeric Ag85B-ESAT-6-IFN- γ protein. This gave it greater immunogenicity compared to the BCG control strain and even compared to recombinant strains expressing these antigens alone [54].

To enhance the vaccine properties of BCG, ESAT-6 is combined with other components related to its expression and secretion, such as proteins of the PE/PPE family. This group is specific for mycobacteria and has similar conserved Pro-Glu and Pro-Pro-Glu motifs, respectively. It includes a large variety of proteins, many of which act as secreted antigens. Some PE and PPE genes are part of the RD1 region and are associated with the ESX secretion system [55].

### 3.2. Ag85 Complex

One of the major virulence factors of Mtb is the Ag85 complex, which consists of three proteins, Ag85A, Ag85B, and Ag85C, encoded by the *fbpA, fbp B,* and *fbpC2* genes, respectively; the major secreted antigen is Ag85B [56]. Ag85 plays an important role in the intracellular survival of Mtb and provokes a strong immune response by inducing IFN-γ and IL-2 cytokine formation in Th1 cells [57]. In addition, Ag85 has an affinity for fibronectin and elastin, which promotes Mtb penetration into macrophages [58]. Additionally, this antigenic complex participates in the formation of the mycobacterial cell wall through mycolyl transferase activity [59]. All these properties make this complex an important factor in the virulence of Mtb. The Ag85 complex is secreted through the conserved Sec export pathway: the primary mode of secretion for many bacteria [60].

A recombinant BCG strain has been engineered that overexpresses the most important Sec proteins SecD, SecF, and SecG [61]. Thus, the level of antigen secretion, particularly Ag85, was elevated. This strain demonstrated increased intracellular survival and persistence in vitro and in vivo and induced enhanced IFN-γ-secreting T cell production. A study of protection against Mtb showed that BCGSecDFG was comparable to BCG. 

The Mtb-related *Mycobacterium smegmatis* serves as a common model for research on mycobacteria, including Mtb, because this organism is nonpathogenic and relatively fast-growing. In addition, attempts are being made on the basis of *M. smegmatis* to create a TB vaccine that can replace BCG. Thus, a recombinant rMs064 vaccine in which *M. smegmatis* expresses epitopes of the Ag85B protein has been considered. There have been several studies of this vaccine [62] in a mouse model and in a J774A.1 mouse macrophage model. Specific humoral and cellular immunogenicity of rMs064, as well as the increased expression of MHC II, CD86, and CD40 and production of IL-1β, TNF-α, IL-12p70, and IL-6, were demonstrated.

The rBCG-Mkan85B vaccine [63] was also based on Ag85B from the mildly pathogenic *Mycobacterium kansasii* microorganism, which has great similarity to Ag85B Mtb. Its use in combination with a booster in the form of a plasmid expressing the Ag85B gene (DNA-Mkan85B) showed an enhanced CD8+ T-cell response to Mtb. This study also identified two novel MHC I (H2-Kd)-restricted epitopes that induce cross-reactivity against mycobacteria, including Mtb.

### 3.3. Protein HspX

The alpha-crystallin protein HspX, a member of the heat shock protein family, is associated with the cell wall. Under the conditions of the lack or absence of oxygen, mycobacteria change and thicken the cell wall, which occurs with the direct participation of HspX. This allows the cells to survive under anaerobic or microaerobic conditions, which is important for penetration into macrophages [64]. This protein is an important antigen that mediates a strong T-cell response leading to IFN-γ and TNF-α secretion [65]. Due to this, the overexpression of the HspX antigen in the BCG vaccine can enhance vaccine efficacy. Notably, HspX is expressed in the latent phase, and such vaccines could potentially be used for prophylaxis in already-infected patients.

A recombinant BCG strain overexpressing the HspX antigen (rBCG::X) was tested in an in vivo model [66]. It expressed not only HspX but also the Ag85B protein. Mice vaccinated with rBCG::X had better protection against Mtb infection, a lower bacterial load in the lungs, and less severe lung pathology compared with the BCG-vaccinated control group.

In addition to recombinant live vaccines, HspX is often used successfully in subunit vaccines, either as a single antigen or in combination with others [67,68,69,70,71]. A study [70] has tested the immunogenicity of a DNA vaccine encoding HspX-PPE44-EsxV fusion antigens alone and in combination with BCG by prime-boost in mice. The expression of these antigens caused increased levels of INF-γ, IL-12, and TGF-β. Thus, the use of these antigens as BCG enhancers may strengthen the immune response.

Another strategy is to modify the interaction between mycobacteria and host cells in order to stimulate the signaling pathways leading to the most effective immune response. Often researchers combine this strategy while expanding the antigenic repertoire of the vaccine to maximize efficacy.

### 3.4. Cyclic di-AMP

Cyclic di-AMP (c-di-AMP) is a widely used secondary messenger in bacteria [72]. It is connected with the regulation of many processes, such as bacterial growth, biofilm formation, potassium transport, and virulence [73]. It also plays a role in immune processes, for example, by mediating an increase in type I interferons’ levels through the activation of the STING-TBK1-IRF3 signaling pathway or IL-1β through the activation of the NLRP3-inflammasome, independent of the STING pathway [74]. It is formed from two ATP molecules by diadenylate cyclase and is cleaved by various phosphodiesterases. For many bacteria, the gene encoding diadenylate cyclase is essential. 

The role of c-di-AMP in Mtb virulence is under discussion, and the data from studies in this area are contradictory. On the one hand, increased c-di-AMP levels have been shown to be associated with the attenuation of Mtb virulence [75]. On the other hand, a later recombinant BCG strain with c-di-AMP overexpression demonstrated an enhanced immune response as well as a longer-lasting effect in a mouse model [76,77]. This is associated with a c-di-AMP-mediated increase in type I IFN production [78].

Recombinant BCG with the overexpression of the endogenous mycobacterial diadenylate cyclase gene *disA* c-di-AMP causes a significant c-di-AMP-mediated increase in IL-6, IL-1β, IFN regulatory factor 3, and IFN-β [79]. In this case, the mechanism of increased production of the interferon gene stimulator STING is involved. The detection of c-di-AMP in the host cytosol leads to the induction of type I interferon via the STING-cGAS signaling pathway, as well as the activation of the NF-κB pathway. The pathogen can influence the intensity of inflammation through the amount of secreted c-di-AMP. Interestingly, the association of high c-di-AMP levels with a stronger Th1 immune response to Mtb infection has also been shown in the *M. smegmatis* model [80].

### 3.5. Mycobacterial Superoxide Dismutase A (SodA) and SecA2

As discussed above, mycobacteria can actively interfere with the intracellular processes of phagocytes and manipulate the immune response. It has been suggested that the lack of efficacy of BCG may be related to its immunosuppressive properties [81].

Deletion of *secA2* in Mtb resulted in partial growth attenuation in vivo. When used as a vaccine, the strain increased the priming of antigen-specific CD8+ T-cells in vivo and reduced bacterial load in the organs of mice and guinea pigs compared with BCG during the Mtb challenge. The authors suggest that an important contribution to the protective properties is made by the preferentially apoptotic type of the death of mutant-infected macrophages. They found out that this effect was dependent on mycobacterial superoxide dismutase A (SodA). SodA is secreted by Mtb through the SecA2 secretion system. It catalyzes the conversion of superoxide anions into hydrogen peroxide. [82]. Reactive oxygen species have a signaling function in activating innate immune responses and influencing the subsequent development of adaptive immunity [83,84]. A BCG strain with the allelic inactivation of the *secA2* and extracytoplasmic-function sigma factor (SigH) was engineered in order to evaluate the role of mycobacterial antioxidants. SigH controls the expression of several antioxidants, including thioredoxin. The modified vaccine had greater immunogenicity than BCG. It demonstrated a greater number of cytokine-producing CD8+ lymphocytes at the peak of the primary immune response and a greater number of IL-2-producing CD4+ lymphocytes [81].

### 3.6. Genes Involved in Phagosome Maturation Delay

The construction of strains deficient in genes whose products are associated with the disruption of phagosome maturation has been suggested as a direction for obtaining new BCG and Mtb vaccine strains.

Zmp1 from Mtb is a virulence factor linked to the stopping of phagosome maturation and preventing inflammasome activation [10]. In addition, recombinant Zmp1 caused the necrotic death of human macrophage-like cells of THP-1, followed by the induction and secretion of necrotic cytokines such as TNF-α, IL-6, and IL-1β as well as chemokines, MCP-1, MIP-1β, and IL-8, indicating its possible role in cell migration and following mycobacterial spreading [85]. It has also been reported that Zmp1 is secreted by Mtb in patients with active TB. This may contribute to disease progression because Zmp1 also stimulates peripheral blood mononuclear cells to release the cytokines that promote a Th2-response that is favorable to the pathogen [86].

The deletion of Zmp1 in BCG affected the bacterial ability to stop phagosome maturation and resulted in the increased presentation of MHC-restricted class II antigens by dendritic cells compared with BCG. The *zmp1* deletion mutant was more immunogenic in the mouse model. The *zmp1* deletion did not affect the survival of BCG-infected mice with severe combined immunodeficiency (SCID) or the growth and distribution of BCG in immunocompetent mice [87]. A protection study of BCG mutants with *zmp1* deletion showed better protection by reducing the bacterial load in the lungs of infected guinea pigs. Additionally, unmarked BCG *zmp1* mutant strains showed a better safety profile in the SCID mouse survival model than the original BCG strains, which is important because HIV is common in regions with a high TB burden as well [88].

Secretory acid phosphatase SapM may be involved in disrupting phagosome-lysosome fusion by removing of phosphatidylinositol 3-phosphate (PI3P) from phagosome membranes which is necessary for the process. The deletion of SapM in Mtb resulted in the increased maturation of Mtb-containing phagosomes in THP-1 cells and decreased virulence in a guinea pig model [9]. However, the transposon disruption of the SapM gene in the BCG strain had no effect on stopping phagosome maturation in the macrophage and dendric cells. Nevertheless, this strain showed improved protectivity in a mouse model of TB infection, which was associated with increased dendric cell recruitment and activation in lymph nodes [89].

### 3.7. Exit of Phagosome

A promising approach to switching the immune response is the obtaining of BCG strains that are capable of penetrating into the cytoplasm of cells. The complementation of BCG with the genes coding ESX-1 that we have already discussed above provides the possibility of the rupture of the phagosome, as well as attaining a response to the immunodominant antigens ESAT-6 and CFP-10. However, an increase in virulence is also possible. To avoid this, a recombinant BCG strain supplemented with ESX-1 of the cold-blooded animal pathogen *Mycobacterium marinum* (BCG::ESX-1Mmar) was constructed. Vaccination with BCG::ESX-1Mmar activated the cGas/STING/TBK1/IRF-3 pathway for type 1 interferon induction and enhanced AIM2 and NLRP3 inflammatory activity, resulting in a higher proportion of specific CD8+ T cells and polyfunctional CD4+ Th1-cells. The strain provided better protection than parental BCG, although the fact that the strain expressed ESX-1-specific immunodominant antigens probably also contributed [90].

The idea of returning BCG to its ability to penetrate cytoplasm appears to be quite successful, as evidenced by the fact that vaccines in which this has been implemented have already participated in clinical trials, and one of them is already in phase III (details below).

## 4. The Most Promising Recombinant Live Vaccines under Clinical Evaluation

We will introduce the most promising recombinant live vaccines under clinical evaluation (Table 1).

### 4.1. AERAS 422

AERAS 422 is a live attenuated BCG-derived vaccine. AERAS 422 cells possess the ability to perforate endosomal membranes due to the expression of PFOG137Q, allowing antigens to penetrate into the cytosol and, thus, stimulating an MHC class I-restricted immune response. PFOG137Q is a gene encoding a mutant perfringolysin (PFOG137Q) from *Clostridium perfringens.* AERAS 422 also contains plasmid encoding immunodominant antigens expressed by Mtb both during active infection (*Rv3804c* and *Rv1886c*, also known as Ag85A and Ag85B, respectively) and during the reactivation of latent infection (*Rv3407*). This vaccine has shown improved protectivity and comparable safety compared to BCG in preclinical studies [91]. However, serious side effects of the vaccine were identified in phase I clinical trials. Two of eight healthy adult volunteers in the trial experienced varicella zoster virus reactivation, and further studies of the vaccine were discontinued [94].

### 4.2. VPM1002

Another TB vaccine currently in clinical trials is rBCG ΔureC::hly, or VPM1002 [92]. It was based on a recombinant BCG strain secreting the listeriolysin (Hly) protein from *Listeria monocytogenes* [95]. This strain has demonstrated a better ability than BCG to stimulate CD8 T-cells [13].

*L. monocytogenes* is one of the few microorganisms that leave the phagosome and enter the cytoplasm, which promotes the presentation of MHC-I-restricted epitopes [96]. An important protein for the exit from the phagosome into the cytoplasm is listeriolysin Hly, which forms a pore. Therefore, its expression in rBCG was expected to increase the efficiency of the CD8 T-cell immune response, which would have a positive effect on the vaccine’s anti-TB qualities.

This strain was further improved by deleting the *ureC* urease gene from the BCG genome. This way, the pH was lowered to an optimal value of 5.5 for Hly, while macrophage apoptosis was also promoted [13]. The resulting rBCG ΔureC::hly vaccine strain demonstrated greater protection than BCG in mouse models, as well as protection against the Beijing strain of MTB, against which BCG does not protect. 

rBCG ΔureC::hly is currently in clinical trials. According to the results of phase I clinical trials conducted in Germany and South Africa, VPM1002 was found to be safe and immunogenic. A randomized Phase IIa clinical trial in healthy infants in South Africa showed that the strain exhibited the necessary immunogenicity in the subjects and was well tolerated. Phase IIb was conducted in healthy and HIV-negative infants [97]. A phase III clinical trial investigating the efficacy of VPM1002 as a prophylaxis for recurrent TB is now planned [98]. 

In addition, a phase III clinical trial involving older adults evaluated the efficacy of this candidate vaccine for the prevention of severe respiratory disease, including COVID-19 [99]. As in previous trials, VPM1002 is well tolerated and appears to have the stated preventive effect against severe respiratory disease (ClinicalTrials.gov: NCT04435379).

### 4.3. MTBVAC

Another example of the vaccine strains in phase III clinical trials is the MTB-derived strain MTBVAC with two inactivated virulence genes. Researchers chose to base the vaccine strain on a clinical isolate from a TB patient, avoiding the use of laboratory-adapted strains such as H37Rv, Erdman, or CDC1551 [93]. The concept of MTBVAC emerged from research into the cause of a deadly outbreak caused by an epidemic strain of *M. bovis*. The *M. bovis* strain is not normally transmitted between humans, but an IS6110 insertion found in the epidemic strain increased the virulence of *M. bovis*. This mutation resulted in increased transcription of the *phoP* gene, which is part of the PhoP/PhoR two-component system [100,101,102]. PhoP has been shown to regulate over 2% of Mtb genes: most of which are associated with virulence [103]. PhoP regulates genes of ESX-1 so that *phoP* mutants can produce but are unable to export ESAT-6 [104].

Other significant virulence genes regulated by PhoP include genes of lipid metabolism (e.g., *pks2, pks3*) involved in the biosynthesis of the polyketide-derived acyl trehalose (DAT, PAT) and sulfolipids. These components, besides their structural function, impede the recognition of Mtb by the immune system [105]. The second gene inactivated in MTBVAC was *fadD26*, a gene of biosynthesis and the export of phthiocerol dimycocerosates (PDIM) which are major virulence-associated lipids of the MTB [106,107]. PDIM has been shown to be connected with phagosomal rupture in concert with ESAT-6 [108,109].

MTBVAC has demonstrated that it is as safe as BCG in preclinical trials [99,110]. In addition, MTBVAC has been shown to provide improved protection compared to BCG [111,112].

Clinical trials of MTBVAC safety began in 2012, after 11 years of preclinical trials. Their result was that intradermal vaccination with MTBVAC was at least as safe as BCG and did not cause serious adverse events. Trials in healthy uninfected newborn infants also confirmed safety and greater immunogenicity compared with the same dose of BCG, suggesting a broader antigenic composition [113].

## 5. Conclusions and Future Directions

This article reviews the molecular mechanisms underlying Mtb–host interactions and the way this knowledge has translated into attempts to create new, more effective vaccines to prevent TB. To summarize, two widespread strategies for creating new TB vaccines have been discussed. The first is the introduction of genes of protective TB antigens and genes affecting antigen presentation. Strains of BCG and other less pathogenic mycobacteria-carrying genes encoding Mtb antigens have been actively developed. Additionally, the attenuated strain of Mtb, MTBVAC, which possesses the entire repertoire of TB antigens, and attenuating mutation different from BCG, is one of the most promising vaccines under clinical evaluation. The second way directs and shifts the interaction with the host cell, such as cell phagosome biology. For example, the integration of the genes providing the phagosome escape led to an increase in the intensity of the immune response. The most successful implementation of this approach has been embodied in a vaccine VPM1002 which is currently in phase III clinical trials.

The division into these two strategies is rather conditional since, in general, both are used in the development of vaccines. This seems to be the best way. However, it has been provided to highlight the areas for further improvement. 

Despite a huge amount of knowledge about BCG and lots of effort to make it perfect, several issues remain unsolved to date. A known disadvantage of BCG is the provoking BCGitises or severe generalized infections in some cases has to be noted. This drawback is present in recombinant BCG strains. Perhaps subunit vaccines solve this problem to a certain extent, but they cannot provide an organism with a wide repertoire of native antigens. Here, it will be worth mentioning one more time the positive nonspecific effects of BCG on immunity, including other diseases, which have already been discussed above. Obviously, subunit vaccines do not have similar properties, which undoubtedly makes them less favorable candidates.

The ideal vaccine to replace BCG should balance the fine line between virulence and protection, providing a potent and long-term immune response to Mtb. The success of clinical trials on vaccines such as MTBVAC and VPM1002 appears to be very promising, but there is still a long road ahead. 

There is a large field for growth in both the above strategies. Identifying antigens associated with latency, as well as cryptic antigens, and amplifying the response to them, seems to be a promising step. The future directions aimed at bringing the creation of a new vaccine closer can be either in returning some antigens into weakened organisms/strains or in further attenuating the virulent ones. It is also necessary to improve our understanding of the action of immunomodulatory and virulent factors that allow Mtb to survive under the pressure of the immune system. A way of influencing these very pathways will permit the retrieval of a vaccine that activates the immune response in a more optimal way. It is not realized in nature due to the evolutionary mechanisms of Mtb.

The combination of these approaches and the use of achievements of genetic engineering may lead to advanced recombinant live vaccines based on BCG, Mtb, or other mycobacteria that will allow the eradication of TB.

## Figures and Tables

**Table 1:** Recombinant live vaccines under clinical evaluation.

Vaccine Name	Main Feature	Reference
AERAS 422	Expression of perfringolysin from *C. perfringens* and antigens Ag85A and Ag85B	[91]
VPM1002	Expression of listeriolysin from *L. monocytogenes* Deletion of the *ureC* gene	[92]
MTBVAC	Mtb with inactivated genes *phoP* and *fadD26*	[93]
